# Cost-effectiveness of 6-year school-based caries preventive interventions in Thailand: a system dynamics modeling study

**DOI:** 10.3389/fpubh.2026.1744986

**Published:** 2026-05-14

**Authors:** Tin Htet Oo, Songchai Thitasomakul, Phongpat Sontamino, Sukanya Tianviwat

**Affiliations:** 1 Department of Preventive Dentistry, Faculty of Dentistry, Prince of Songkla University, Songkhla, Thailand; 2 Department of Mining and Materials Engineering, Faculty of Engineering, Prince of Songkla University, Songkhla, Thailand

**Keywords:** children, cost-effectiveness, sealant, system dynamics model, toothbrushing

## Abstract

**Objective:**

This study evaluated the cost-effectiveness of 6-year school-based caries preventive interventions [supervised toothbrushing (STB) and dental sealant] in Thailand using a System Dynamics Model (SDM).

**Methods:**

An SDM, which simulates changes in population health over time using stocks, flows, and feedback relationships, was developed to represent caries progression. In total, three scenarios were analyzed: STB, sealant, and a base case without intervention for 6 years, covering children aged 6–12 years. Costs included interventions and treatment (fillings, endodontic treatment, and extraction). Effectiveness was measured as the number of caries-free children. Model inputs were derived from national data, literature, and expert validation. Changes in outcomes and costs were driven by intervention effects.

**Results:**

Sealant had a lower cost-effectiveness ratio (CER) of 505.3 Thai baht (15.79 USD) per caries-free child versus 537.2 baht (16.79 USD) for STB. Both were more costly and more effective than the base case, with an incremental effectiveness ratio (ICER) of 1,985.3 baht (62.04 USD) for STB and 1,210.5 baht (37.83 USD) for sealant per additional caries-free child. Sealant was dominant over STB (negative ICER), meaning it was more effective and less costly. In best-case sensitivity scenarios, sealant was more effective but also more costly than STB, representing a trade-off, with an ICER of 121.54 baht (3.80 USD) per additional caries-free child.

**Conclusion:**

Sealant is the preferred strategy or a trade-off option when resources allow for additional caries-free children, while STB can serve as a low-cost alternative. Policy should prioritize sealant by optimizing effectiveness, controlling costs, and targeting high-risk groups.

## Introduction

Globally, untreated dental caries in permanent teeth is the most common health condition, affecting an estimated 2 billion people (1). In Thailand, dental caries remains a major public health concern among school-aged children. National Oral Health Surveys report that 52.0% of 12-year-olds and 62.7% of 15-year-olds are affected by dental caries (2). Untreated caries negatively affects oral health-related quality of life (OHRQoL), resulting in pain, difficulty chewing, poor appetite, and impaired school performance (3). These conditions often require treatment; however, high service costs and inequalities in access to dental care remain major concerns (4, 5).

Preventive strategies implemented during childhood are essential for reducing disease burden and reducing disparities in dental services utilization. Evidence from previous studies in Thailand indicates that preventive interventions, such as supervised toothbrushing (STB) and dental sealant application, can effectively reduce the incidence of dental caries among children (6, 7). As a result, the Ministry of Public Health (MOPH) in Thailand has implemented national preventive programs targeting children aged 6–12 years, including supervised toothbrushing and dental sealant programs in schools (8).

Policymakers require economic evidence to determine whether the interventions represent efficient use of public health resources. Most economic evaluations have applied modeling techniques, such as decision trees or Markov models, which typically assume fixed transition probabilities and limited feedback interactions (9, 10). System Dynamics Model (SDM) provides an alternative approach for evaluating complex health systems (11–13). SDM is a computer-based simulation designed to analyze how system components interact over time through feedback loops and time delays (11–13). In the context of oral health, SDM can capture the dynamic interactions between caries development, treatment utilization, preventive interventions, and oral health outcomes by exploring different scenarios. This approach allows researchers and policymakers to assess the potential impacts of interventions under different policy conditions.

In Thailand, previous cost studies have reported average costs per patient for a variety of dental services (4, 14), while some economic evaluations have assessed the cost-effectiveness of caries prevention strategies, particularly dental sealant and school-based oral health programs in different settings (15–18). A previous study in Thailand applied a System Dynamics Model (SDM) to estimate the long-term effects of supervised toothbrushing and dental sealant programs implemented by the Ministry of Public Health (MOPH) for children aged 6–12 years (19); however, it did not incorporate cost data or evaluate the economic implications of these interventions. In particular, none of the existing cost studies have examined dental care costs under different intervention scenarios over time using SDM. Consequently, there remains a knowledge gap regarding whether these preventive interventions represent an efficient use of healthcare resources within the Thai public health system. To address this gap, the present study aims to evaluate the cost-effectiveness of 6-year school-based caries preventive interventions: supervised toothbrushing (STB) and dental sealant programs implemented by the MOPH for children using SDM. The analysis incorporates both intervention costs and treatment costs associated with dental caries, including fillings, endodontic treatment, and tooth extraction, from a provider perspective. The effectiveness outcome is defined as the number of caries-free children under three scenarios: supervised toothbrushing, dental sealant application, and a base case scenario without intervention. This outcome was selected because it directly reflects the primary goal of caries preventive programs and is easily interpretable for policymakers as a national oral health indicator commonly used in Thailand (2). By integrating economic evaluation with the System Dynamics Model, this study aims to provide evidence for policymakers and public health decision-makers in the efficient use of resources for oral health prevention programs.

Therefore, the objective of this study was to evaluate the cost-effectiveness of 6-year school-based caries preventive interventions [supervised toothbrushing (STB) and dental sealants] implemented by the Ministry of Public Health (MOPH) in Thailand, targeting children aged 6–12 years.

## Materials and methods

### Caries preventive interventions

#### Supervised toothbrushing (STB)

Supervised toothbrushing (STB) is a national oral health promotion program implemented by the Ministry of Public Health (MOPH), Thailand, targeting children aged 6–12 years (8). The program aims to reduce the risk of dental caries by promoting regular toothbrushing with fluoridated toothpaste and reinforcing proper oral hygiene practices among school-aged children (8, 20). In school settings, teachers receive training or guidance on the correct toothbrushing technique from dental professionals. Children are supervised by teachers to brush their teeth twice daily using fluoridated toothpaste and age-appropriate toothbrushes, following the correct toothbrushing technique recommended by dental professionals during school days. In some settings, parents follow the guidance and participate in supervising toothbrushing at home to reinforce the behavior established at school.

#### Dental sealant

The Ministry of Public Health (MOPH), Thailand provides dental sealant application as a preventive service for children aged 6–12 years (8). The program targets to protect caries development in permanent teeth due to deep pits and fissures (8, 21). In practice, dental sealants are applied during school dental visits conducted by dental professionals from local hospitals or community health centers. During these visits, resin-based sealants are applied to the occlusal surfaces of permanent teeth to prevent the accumulation of food debris and bacterial plaque within pits and fissures.

### Base case

The base case scenario assumes the situation in which children are not covered by the supervised toothbrushing (STB) program or the dental sealant intervention supported by the Ministry of Public Health (MOPH), Thailand. Under the base case, children may practice routine toothbrushing based on their individual or household habits. The frequency and quality of toothbrushing and usage of toothpaste may vary among individuals, but it is not promoted through public health programs. In addition, no professional dental sealant applications are provided. Therefore, the development and progression of dental caries in the base case are assumed to follow the natural course of disease development influenced by existing behavioral factors without intervention support, and caries cases under this scenario may receive treatment through routine dental services when available.

### Treatment for caries

Caries treatment refers to clinical procedures aimed at managing dental caries to prevent further disease progression and to restore oral health (22). In this study, caries treatment procedures include dental filling, endodontic treatment, and extraction of carious teeth. Children aged 6–12 years who developed carious lesions and sought dental treatment were considered the caries treatment population in this study.

### System dynamics model (SDM)

**Figure 1:**
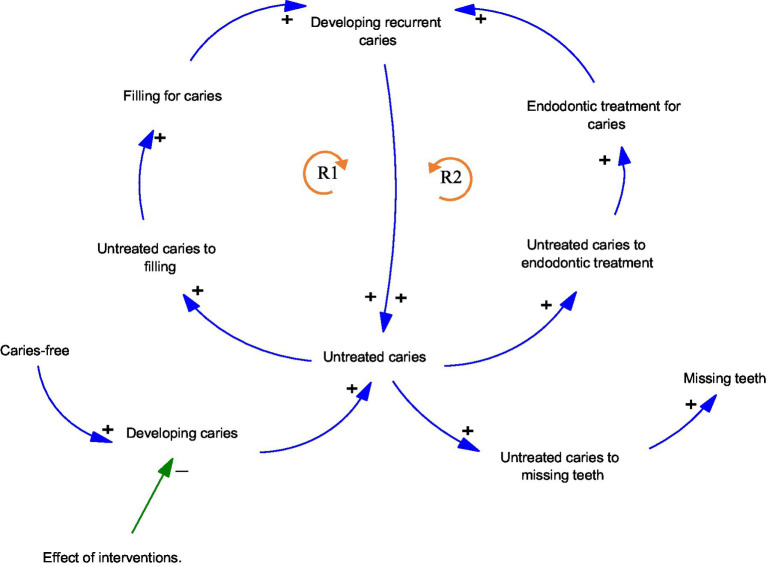
Causal loop diagram (19). (+) indicates an increase in one variable leads to an increase in the related variable and a decrease leads to a decrease. (−) indicates an increase in one variable leads to a decrease in another variable and vice versa. R1 and R2 represent reinforcing feedback loops where an increase in one variable leads to increases in related variables through feedback. When interventions are applied, a decrease in one variable leads to decreases in related variables through feedback.

**Figure 2:**
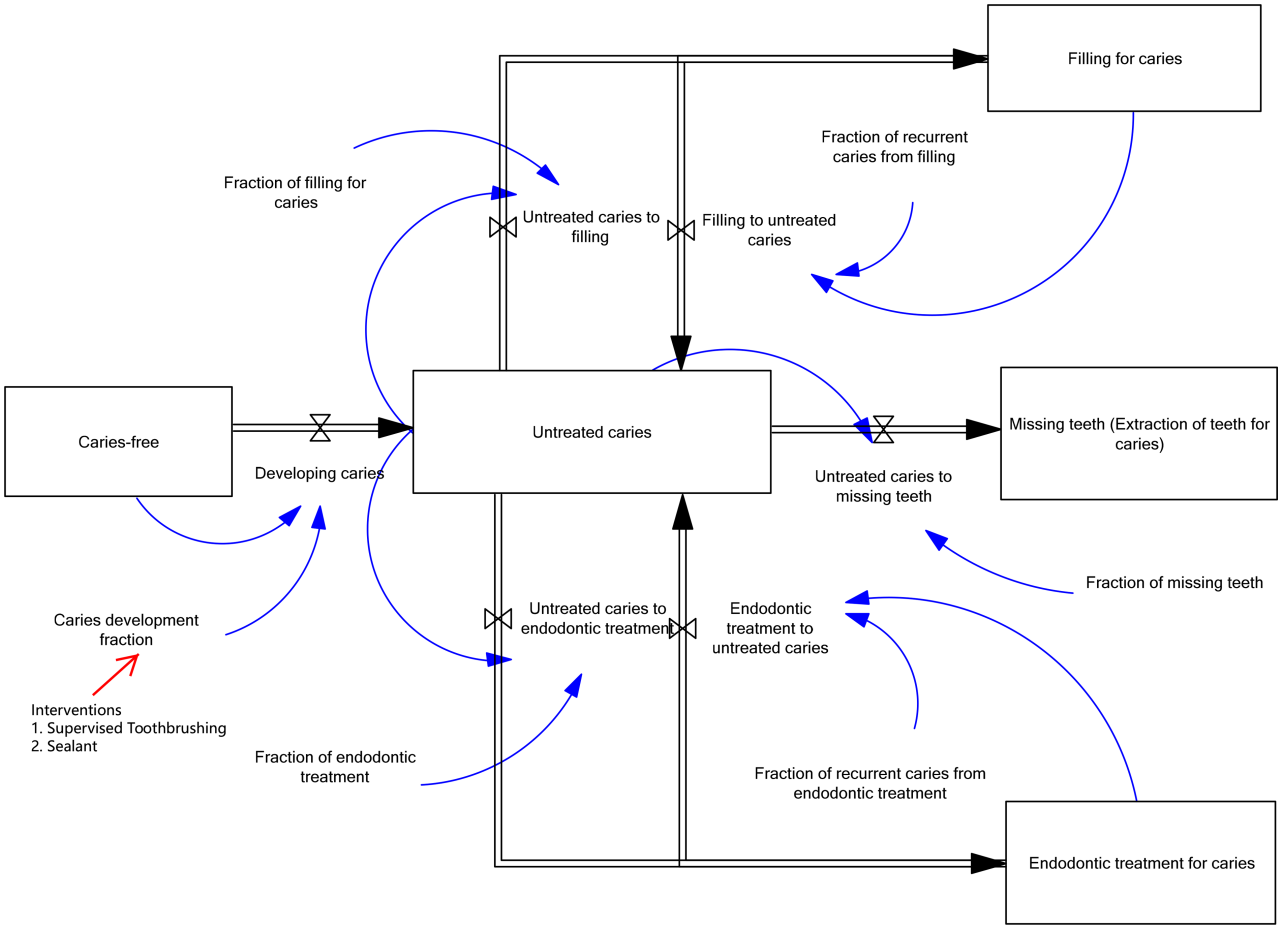
Stocks and flows diagram (19). Square blocks 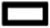: Stocks (represent health state in the model such as caries-free, caries, treated cases, and tooth loss). Straight arrows 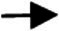: Flows (control the changes in stocks). Blue circular arrows 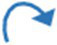: Feedback loops, parameters, or fractions that control flows.

SDM was developed through Group Model Building (GMB) sessions. The sessions involved experts in related fields and researchers. In the sessions, the conceptual structure of the SDM was illustrated by the causal loop diagram (CLD) that describes the relationships among key variables related to dental caries progression and treatment (Figure 1). The diagram represents the interactions between no caries, caries development, untreated caries, treated caries, recurrent caries, and tooth loss. The model assumption is that children initially enter the system in the no caries state and over time, some individuals transit to caries development due to exposure to risk factors, which may lead to untreated caries. If untreated, the disease can progress and eventually result in tooth loss. Children with untreated caries may also receive dental treatment, including restorative treatment (dental filling) or endodontic treatment. The CLD includes two reinforcing feedback loops representing the progression of caries and its consequences. The first reinforcing loop (R1) shows that an increase in untreated caries leads to more restorative treatments, and treated teeth are more susceptible to recurrent caries, which may return to the untreated caries state and further increase untreated cases. The second reinforcing loop (R2) represents that an increase in untreated caries leads to more endodontic treatments. It may also develop recurrent caries, which can again contribute to untreated caries over time. The implementation of supervised toothbrushing (STB) and dental sealant programs lowers the rate of caries development, leading to fewer untreated caries cases and consequently, treated cases, recurrent caries, and tooth loss are also reduced, counteracting the reinforcing dynamics of the system. The CLD was converted to a stocks and flows diagram as shown in Figure 2. The square blocks in the figure represent the stocks to quantify the outcomes, such as the populations with caries-free, caries, filling for caries, endodontic treatment for caries, and missing teeth (extraction of teeth for caries). The stocks are varied by inflows and outflows, denoted by the arrows. The fractions control the rate of flow. The effectiveness percentages of interventions reduce caries fraction, by using the formula caries fraction = Base case caries fraction × (1 − effectiveness rate in %/100), thereby the rate of flow of developing caries was reduced compared with the base case. The model was run to identify the outcomes by comparing intervention scenarios (STB and sealant) and the base case. Structural validity and behavioral validity for the model were assessed (23, 24). The model’s structural validity was assessed by determining the model boundaries, the model’s structures, parameters, and the dimensional consistency of its equations during GMB sessions. Behavior validity was assessed by comparing the simulated caries-related outcomes with historical data. Since the research started in 2022, Thais who were born in 2021 and registered in Thailand’s National Statistical Office were set in the model. The total population was 678,243. The background characteristics and behaviors of the study population were assumed to be similar, representing the current conditions in Thailand (2). The model simulated this population, assuming a starting age of 6 years up to 12 years. This is the age period during which interventions are provided. Vensim PLE version 6.4 software was used to run the model. Parameter values are provided in Supplementary Table S1.

**Figure 3:**
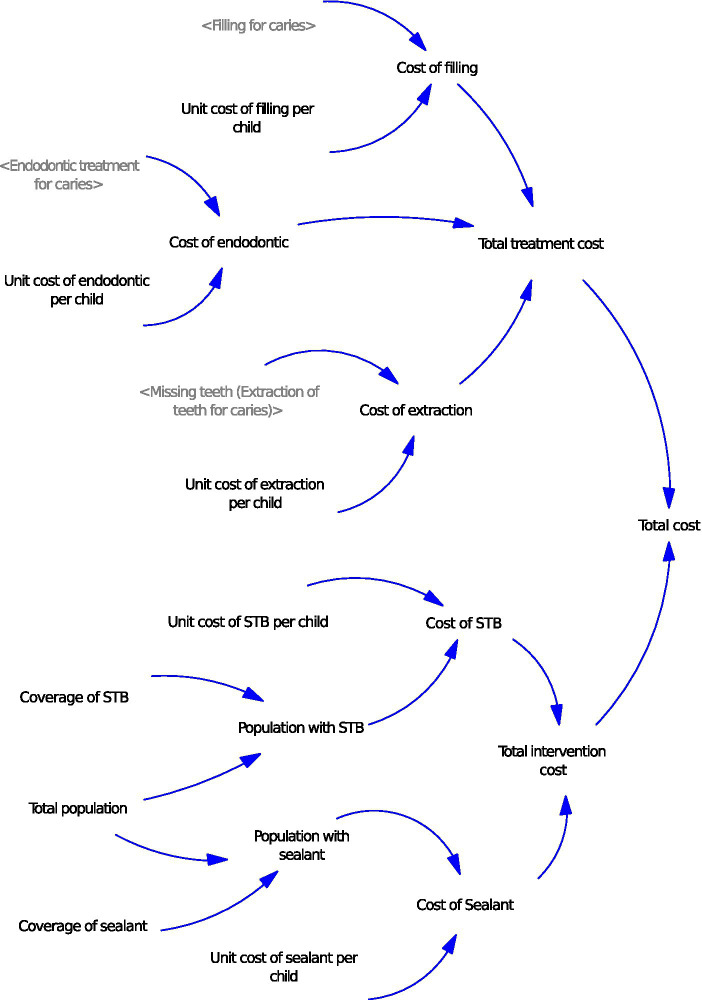
Cost variables STB: Supervised toothbrushing. Arrows represent relationships between variables. Treatment costs (filling, endodontic treatment, and extraction) are calculated from the number of treated cases and unit cost per child. Treated cases are came from Figure 2. Intervention costs (supervised toothbrushing and sealant) are estimated using coverage and unit cost per child. Total treatment cost and total intervention cost are summed for the total cost.

The present study estimated the costs based on those outcome populations. The cost variables can be seen in Figure 3. The costs of filling, endodontic treatment, and extraction for caries were estimated based on the number of individuals receiving these treatments, as simulated from the stocks and flows diagram (Figure 2), and the unit cost of each treatment. The costs of supervised toothbrushing (STB) and sealant interventions were estimated based on the unit cost of each intervention and their respective coverage levels.

### Data sources

The input data for the stocks, flows, and fractional variables in Figure 2 were obtained from secondary data sources, including the 8th National Oral Health Survey (NOHS) of Thailand (2), the National Statistical Office (25), the Health Data Center (HDC) (26), and relevant published literature (19, 27–31). These sources were selected because they provide relevant information on oral health conditions and service utilization in Thailand. Coverage rates of sealant and STB programs were obtained from the Health Data Center (HDC) of Thailand for 2022 and Thai literature (26, 29). They were selected because they are consistent with national data and reflect the context of the Thai healthcare system. The effectiveness rates of preventive interventions were derived from a systematic review and meta-analysis that was conducted by researchers to ensure relevance and methodological rigor (19, 32). Cost parameters for supervised toothbrushing (STB), dental sealant, and caries treatments (filling, endodontic treatment, and extraction) were collected from Thailand studies (14, 33–38), which reflect the local healthcare context. However, as the model relies primarily on secondary data, potential limitations such as reporting bias, missing data, and differences in data collection methods across sources were considered during the parameter selection process. All parameter values were compiled in an Excel spreadsheet and reviewed by experts during Group Model Building (GMB) sessions to assess their relevance and validity for the model.

### Scenarios

Intervention scenarios represent the caries preventive interventions, STB, and sealant provided by the MOPH, Thailand for 6–12 years. The effectiveness rate of STB was 10% and so 9.5% in coverage of 95% (32). While the sealant was 58.0%, and thus 15.7% in coverage of 27% (32). Under the intervention scenarios, the caries fraction was reduced by applying the effectiveness rates of STB and sealant, calculated as: caries fraction = base case caries fraction × (1 – effectiveness rate in %/100) (32). This reduction in the caries fraction led to a decrease in the annual rate of caries development. The base case scenario was set as a reference to compare with intervention scenarios. It was assumed that the population was not covered by interventions under the base case scenario. No parameters were changed, and the rate of caries development was not affected by the effect of the intervention in the base case. Under the STB scenario, the cost of STB, treatment costs after STB implementation, and total cost were assessed. Similarly, the cost of sealant, treatment costs after application of sealant, and total cost were analyzed under the sealant scenario. No intervention was charged in the base case, and only treatment costs were estimated in the base case scenario.

### Cost information

Cost data were identified through a structured manual search of studies conducted in Thailand (2008–2025), focusing on context-specific estimates for dental services. Sources included national dental public health journals, health policy journals, faculty-based journals from major Thai dental schools, and relevant academic theses, as well as peer-reviewed articles indexed in PubMed. A manual approach was used to capture limited indexing of Thai-language journals and grey literature.

Studies were included if they reported cost data for dental services in Thailand using a clearly described and methodologically sound approach. Eligible studies provided primary cost data from real service settings. Cost components needed to include key inputs such as personnel (e.g., salaries), materials (e.g., consumables and dental materials), and, where applicable, capital costs (e.g., equipment, annualized or depreciated), and, where available, overhead or indirect costs, and describe their costing methods transparently. Preference was given to studies conducted in the public sector or routine service settings in Thailand.

Cost estimates, study year, perspective, and cost components were extracted from each study (14, 33–38), and all costs were converted to Thai baht. To ensure comparability, costs were adjusted to 2024 price levels using the Thai Consumer Price Index (CPI; base year 2019 = 100) from the Bureau of Trade and Economic Indices (39).


$${\text{Adjusted Cost}}_{2024}={\text{Cost}}_{\text{year}}\times \frac{\mathrm{CP}I_{2024}}{\mathrm{CP}I_{\text{year}}}$$

This standardization expresses all costs in real terms for valid comparison across studies.

Most studies reported only point estimates, and meta-analysis was not feasible. A pragmatic synthesis was applied, with all costs converted to Thai baht and adjusted to 2024 values using the Thai CPI (base year 2019). Costs were summarized by mean, minimum, and maximum. The unweighted mean was used for the base case, while minimum and maximum values were used to reflect uncertainty in sensitivity analyses.

The costs of interventions (STB and sealant) and the costs of treatment (filling, endodontic, and extraction) were involved in this study. After adjusting with CPI, the average unit cost of STB was estimated at 57.6 Thai baht per child. In the model (Figure 3), the cost of STB was calculated by multiplying the unit cost of the STB program by the population that was covered by the program. The population implemented by STB came from multiplying the coverage of STB by the total population. The unit cost of sealant per child was 203.5 baht. The cost of sealant in the model (Figure 3) was set by multiplying the unit cost of sealant by the population that received sealant application. The population with sealant was obtained by multiplying the coverage of sealant by the total population. The unit costs of filling, endodontic treatment, and tooth extraction were estimated at 360.1 baht, 1839.4 baht, and 133.6 baht per child, respectively. The costs of filling, endodontic, and extraction were figured by multiplying the unit cost of each treatment per child by the treated population, as shown in Figure 3. The population that experienced treatment came from the stocks in Figure 2, representing the population with filling for caries, endodontic treatment for caries, and missing teeth (extraction of teeth for caries).

### Cost-effectiveness analysis

The analysis included the cost-effectiveness ratio (CER) and incremental cost-effectiveness ratio (ICER). The cost-effectiveness ratio (CER) involves dividing the cost of an intervention by its effectiveness (40). The incremental cost-effectiveness ratio (ICER) is calculated by dividing the difference in costs between two interventions or intervention and comparator (base case) by the difference in their effects (40). The cost was the total cost, including the intervention costs and treatment costs, and the effectiveness was the caries-free population. Both the total cost and caries-free population under STB, sealant, and base case were estimated by the SDM as mentioned above. The CER of STB, sealant, and the base case were analyzed and compared among them. The base case was used as a reference for ICER to compare interventions.

### Sensitivity analysis

A multi-way sensitivity analysis was performed to examine changes in the total cost, CER, and ICER under STB and sealant scenarios by varying key parameters: coverage, effectiveness rates, and unit costs. The base case was used as a reference, with parameters unchanged. Effectiveness rates and unit costs were set at their maximum and minimum values, while coverage was set at current and maximum levels for both STB and sealant scenarios. The maximum coverage was 95% for STB and 47.2% for sealant, representing the national targets in Thailand (29), while current coverage was 27% for sealant and 95% for STB (26, 29). The maximum effectiveness rates were 21% for STB and 82% for sealant, and the minimum were 0% for STB and 52% for sealant. These values were retrieved from the upper and lower bounds of systematic reviews and meta-analyses (32). All effectiveness rates were adjusted for coverage. Unit costs were varied as follows (Thai Baht): filling 270.2–471.5, endodontic treatment 1,315.7–2,619.7, extraction 100.5–155.9, STB 34.8–80.4, and sealant 92.2–366.8. These values were derived from the cost calculations described above.

## Results

Table 1 presents the comparison of costs under three scenarios: base case, supervised toothbrushing (STB), and sealant over time. Total intervention cost for sealant was slightly higher than STB across years. However, treatment costs were consistently lower for both interventions than the base case, reflecting reduced disease burden. At 12 years, total costs were 104.6 million Thai baht (3.27 million USD) for STB and 103.9 million baht (3.25 million USD) for sealant, compared to 68.9 million baht (2.15 million USD) in the base case. Between the two interventions, sealant had a slightly lower total cost than STB.

**Table 1:** Comparison of costs under three scenarios.

Year	Total intervention cost in million		Total treatment cost in million			Total cost in million		
	Thai baht (USD)		Thai baht (USD)			Thai baht (USD)		
	STB	Sealant	Base case	STB	Sealant	Base case	STB	Sealant
6	37,113,500 (1.16)	37,266,100 (1.16)	39,857,300 (1.25)	39,857,300 (1.25)	39,857,300 (1.25)	39,857,300 (1.25)	76,970,800 (2.41)	77,123,400 (2.41)
7	37,113,500 (1.16)	37,266,100 (1.16)	42,490,200 (1.33)	42,490,200 (1.33)	42,490,200 (1.33)	42,490,200 (1.33)	79,603,700 (2.49)	79,756,300 (2.49)
8	37,113,500 (1.16)	37,266,100 (1.16)	46,440,500 (1.45)	46,277,900 (1.45)	46,184,900 (1.44)	46,440,500 (1.45)	83,391,400 (2.61)	83,451,000 (2.61)
9	37,113,500 (1.16)	37,266,100 (1.16)	51,330,200 (1.60)	50,903,900 (1.59)	50,658,300 (1.58)	51,330,200 (1.60)	88,017,400 (2.75)	87,924,400 (2.75)
10	37,113,500 (1.16)	37,266,100 (1.16)	56,855,600 (1.78)	56,111,000 (1.75)	55,678,300 (1.74)	56,855,600 (1.78)	93,224,500 (2.91)	92,944,400 (2.90)
11	37,113,500 (1.16)	37,266,100 (1.16)	62,775,200 (1.96)	61,691,200 (1.93)	61,056,200 (1.91)	62,775,200 (1.96)	98,804,700 (3.09)	98,322,300 (3.07)
12	37,113,500 (1.16)	37,266,100 (1.16)	68,898,100 (2.15)	67,478,000 (2.11)	66,639,100 (2.08)	68,898,100 (2.15)	104,591,500 (3.27)	103,905,200 (3.25)

STB, supervised toothbrushing; USD, United States Dollar. Total cost = Total intervention cost + Total treatment cost. The base case has no intervention cost as no intervention was implemented. Costs are presented in Thai baht and USD using an exchange rate of 1 USD = 32 baht (March 2026).

Table 2 presents the cost-effectiveness results of the base case, supervised toothbrushing (STB), and sealant. The cost-effectiveness ratio (CER) was lowest in the base case [389.9 Thai baht (12.18 USD) per caries-free child], followed by sealant [505.3 baht (15.79 USD)], and highest in STB [537.2 baht (16.79 USD)]. In terms of incremental cost-effectiveness ratio (ICER), both interventions were more costly and more effective than the base case, with ICERs of 1,985.3 baht [62.04 USD] for STB and 1,210.5 baht [37.83 USD] for sealant per additional caries-free child. When comparing sealant to STB, sealant was more effective and less costly than STB, and is dominant (cost-saving) [ICER: −62.7 baht (−1.96 USD)].

**Table 2:** Incremental cost-effectiveness ratio (ICER) compared among three scenarios at 12 years old.

Scenario	Total cost in million	Caries-free population	CER in Thai baht (USD)	ICER in Thai baht (USD)	ICER in Thai baht (USD)
	Thai baht (USD)			Interventions and base case	STB and sealant
Base case	68,898,100 (2.15)	176,722	389.9 (12.18)	-	-
STB	104,591,500 (3.27)	194,701	537.2 (16.79)	1985.3 (62.04)	-
Sealant	103,905,200 (3.25)	205,642	505.3 (15.79)	1210.5 (37.83)	−62.7 (−1.96) [dominant]

CER, cost-effectiveness ratio; ICER, incremental cost-effectiveness ratio; STB, supervised tooth brushing; USD, United States Dollar. CER = Total cost ÷ Caries-free population. ICER interventions and base case = (Total cost of intervention − Total cost of base case) ÷ (Caries-free of intervention − Caries-free of base case). ICER STB and sealant = (Total cost of STB − Total cost of sealant) ÷ (Caries-free of STB − Caries-free of sealant). A negative ICER indicates that the sealant strategy is less costly and more effective than STB (i.e., dominant). Costs are presented in Thai baht and USD using an exchange rate of 1 USD = 32 baht (March 2026); effectiveness is measured as the number of caries-free children.

Table 3 shows the sensitivity analysis for STB by varying coverage, effectiveness, and unit costs, using the base case as the reference. CER ranged from 333.1 to 804.3 baht (10.41–25.13 USD) per caries-free child. The lowest CER was observed in scenarios 3 and 7 [333.1 baht (10.41 USD)], while the highest CER was in scenario 2 [804.3 baht (25.13 USD)]. For ICER, scenarios with no effectiveness gain (1, 2, 5, and 6) had undefined values. Scenarios 3 and 7 had the lowest ICER [64.6 baht (2.02 USD) per additional caries-free child], whereas scenarios 4 and 8 had much higher ICERs [1,854.6 baht (57.96 USD)]. Scenario 7 was considered the best-case scenario with maximum coverage, maximum effectiveness, and minimum unit costs. Scenario 2 was considered the worst-case scenario with minimum coverage, minimum effectiveness, and maximum unit costs.

**Table 3:** Multi-way sensitivity analysis for supervised toothbrushing (STB) at 12 years old.

Scenarios		Coverage	Effectiveness	Unit costs	Total cost in million	Caries-free population	CER in Thai baht (USD)	ICER in Thai baht (USD)
					Thai baht (USD)			
STB	1	Current	Min	Min	73,473,400 (2.30)	176,722	415.8 (12.99)	NA
	2^#^	Current	Min	Max	142,128,400 (4.44)	176,722	804.3 (25.13)	NA
	3	Current	Max	Min	71,316,400 (2.23)	214,179	333.1 (10.41)	64.6 (2.02)
	4	Current	Max	Max	138,365,700 (4.32)	214,179	646.3 (20.20)	1854.6 (57.96)
	5	Max	Min	Min	73,473,400 (2.30)	176,722	415.8 (12.99)	NA
	6	Max	Min	Max	142,128,400 (4.44)	176,722	804.3 (25.13)	NA
	7^*^	Max	Max	Min	71,316,400 (2.23)	214,179	333.1 (10.41)	64.6 (2.02)
	8	Max	Max	Max	138,365,700 (4.32)	214,179	646.3 (20.20)	1854.6 (57.96)
Base case		-	-	-	68,898,100 (2.15)	176,722	389.9 (12.18)	-

CER, cost-effectiveness ratio; ICER, incremental cost-effectiveness ratio; STB, supervised tooth brushing; Min, minimum; Max, maximum; USD, United States Dollar; NA, not applicable. CER = Total cost ÷ Caries-free population. ICER = (Total cost of STB − Total cost of base case) ÷ (Caries-free of STB − Caries free of base case). Positive ICER: cost and effectiveness differences are in the same direction (either higher cost and higher effect, or lower cost and lower effect); ICER is calculated only when STB improves effectiveness; otherwise, it is an undefined value, “not applicable.” The base case is set as a reference without changing parameters. Costs are presented in Thai baht and USD using an exchange rate of 1 USD = 32 baht (March 2026); effectiveness is measured as the number of caries-free children. ^*^Best case = scenario 7; ^#^Worst case = scenario 2.

Table 4 describes the sensitivity analysis for sealant by varying coverage, effectiveness, and unit costs, using the base case as the reference. CER ranged from 299.4 to 910.9 baht (9.36–28.47 USD) per caries-free child. The lowest CER was observed in scenario 7 [299.4 baht (9.36 USD)], while the highest CER occurred in scenario 6 [910.9 baht (28.47 USD)]. Scenarios 1 and 3 were dominant (cost-saving) compared to the base case [ICER: −95.7 baht (−2.99 USD) and −80.4 baht (−2.51 USD)]. Scenario 7 had a low positive ICER [94.2 baht (2.94 USD) per additional caries-free child], representing the best-case scenario with maximum coverage, maximum effectiveness, and minimum unit costs. Scenario 2 had the highest ICER [3,342.6 baht (104.46 USD)], reflecting the worst-case scenario with minimum coverage, minimum effectiveness, and maximum unit costs.

**Table 4:** Multi-way sensitivity analysis for sealant at 12 years old.

Scenarios		Coverage	Effectiveness	Unit costs	Total cost in million	Caries-free population	CER in Thai baht (USD)	ICER in Thai baht (USD)
					Thai baht (USD)			
Sealant	1	Current	Min	Min	66,436,500 (2.08)	202,445	328.12 (10.25)	−95.7 (−2.99)
	2^#^	Current	Min	Max	154,880,800 (4.84)	202,445	765.1 (23.91)	3342.6 (104.46)
	3	Current	Max	Min	65,535,800 (2.05)	218,558	299.9 (9.37)	−80.4 (−2.51)
	4	Current	Max	Max	153,309,600 (4.79)	218,558	701.5 (21.92)	2017.7 (63.05)
	5	Max	Min	Min	77,923,300 (2.43)	223,010	349.4 (10.92)	194.9 (6.09)
	6	Max	Min	Max	203,137,000 (6.35)	223,010	910.9 (28.47)	2900.1 (90.63)
	7^*^	Max	Max	Min	76,237,400 (2.38)	254,669	299.4 (9.36)	94.2 (2.94)
	8	Max	Max	Max	200,196,000 (6.26)	254,669	786.1 (24.57)	1684.5 (52.64)
Base case		-	-	-	68,898,100 (2.15)	176,722	389.9 (12.18)	-

CER, cost-effectiveness ratio; ICER, incremental cost-effectiveness ratio; Min, minimum; Max, maximum; USD, United States Dollar. CER = Total cost ÷ Caries-free population. ICER = (Total cost of sealant − Total cost of base case) ÷ (Caries free of sealant − Caries free of base case). Positive ICER: cost and effectiveness differences are in the same direction (either higher cost and higher effect, or lower cost and lower effect); negative ICER: cost and effectiveness differences are in opposite directions (e.g., higher cost and lower effect, or lower cost and higher effect). The base case is set as a reference without changing parameters. Costs are presented in Thai baht and USD using an exchange rate of 1 USD = 32 baht (March 2026); effectiveness is measured as the number of caries-free children. ^*^Best case, scenario 7; ^#^Worst case, scenario 2.

Table 5 shows the comparison of the best and worst-case scenarios between STB and sealant. In the best and worst comparison, STB (best) had a lower CER [332.9 baht (10.40 USD)] than sealant (worst) [765.1 baht (23.91 USD)] and was dominant [ICER: −7,121.56 baht (−222.55 USD)]. In the best and best comparison, sealant (best) had a lower CER [299.4 baht (9.36 USD)] than STB (best) [332.9 baht (10.40 USD)]; however, the ICER [121.54 baht (3.80 USD)] shows that sealant incurs a small additional cost per additional caries-free child compared to STB. In the worst and worst comparison, sealant (worst) showed a lower CER [765.1 baht (23.91 USD)] than STB (worst) [804.3 baht (25.13 USD)], suggesting better efficiency despite higher total cost. The ICER [495.76 baht (15.49 USD)] indicates additional cost for improved effectiveness. In the worst and best comparison, sealant (best) was dominant compared to STB (worst), with a lower CER [299.4 baht (9.36 USD) and 804.3 baht (25.13 USD)] [ICER: −845.33 baht (−26.42 USD)].

**Table 5:** Comparison of best-case and worst-case scenarios of supervised toothbrushing (STB) and sealant from multi-way sensitivity analysis.

Scenario comparison	Intervention	Total Cost in million	Caries-free population	CER in Thai baht (USD)	ICER in Thai baht (USD)
		Thai baht (USD)			
Best and worst	STB (best)^*^	71,316,400 (2.23)	214,179	332.9 (10.40)	−7,121.56 (−222.55)
	Sealant (worst)^#^	154,880,800 (4.84)	202,445	765.1 (23.91)	–
Best and best	STB (best)^*^	71,316,400 (2.23)	214,179	332.9 (10.40)	121.54 (3.80)
	Sealant (best)^*^	76,237,400 (2.38)	254,669	299.4 (9.36)	–
Worst and worst	STB (worst)^#^	142,128,400 (4.44)	176,722	804.3 (25.13)	495.76 (15.49)
	Sealant (worst)^#^	154,880,800 (4.84)	202,445	765.1 (23.91)	–
Worst and best	STB (worst)^#^	142,128,400 (4.44)	176,722	804.3 (25.13)	−845.33 (−26.42)
	Sealant (best)^*^	76,237,400 (2.38)	254,669	299.4 (9.36)	–

CER, cost-effectiveness ratio; ICER, incremental cost-effectiveness ratio; STB, supervised tooth brushing; Min, minimum; Max, maximum; USD, United States Dollar. CER = Total cost ÷ Caries-free population. ICER = (Total cost of STB − Total cost of sealant) ÷ (Caries-free of STB − Caries free of sealant). Positive ICER: cost and effectiveness differences are in the same direction (either higher cost and higher effect, or lower cost and lower effect); negative ICER: cost and effectiveness differences are in opposite directions (e.g., higher cost and lower effect, or lower cost and higher effect). Costs are presented in Thai baht and USD using an exchange rate of 1 USD = 32 baht (March 2026); effectiveness is measured as the number of caries-free children. ^*^Best case = scenario 7; ^#^Worst case = scenario 2.

## Discussion

The model demonstrated that children transition from the no-caries state to the caries development state, treated state, recurrent disease state, and tooth loss over time, where the rate of caries development was reduced by implementing STB and sealant programs compared to the base case. The model assumes that preventive interventions increase the caries-free population, thereby reducing treatment needs, and changes in costs were driven by intervention effects. On the other hand, it did not account for socioeconomic or behavioral factors specifically and relies on secondary data for cost and effectiveness parameters, which may introduce structural and parameter uncertainty. However, multi-way sensitivity was performed by varying the key parameters such as coverage, effectiveness rates, and unit costs to better reflect uncertainty in key inputs.

The previous study showed that the sealant application was more efficacious than STB in preventing dental caries, with the model showing a 25.5% increase in the caries-free population compared to 14.5% with STB (19). The present study aligns with previous findings that sealant provides the lowest treatment cost for children. Given the high treatment expenses in Thailand, preventive interventions can reduce disease burden and future treatment needs, even though they require investment. Both interventions were more costly and more effective than the base case. Between the two interventions, sealant was more effective and slightly less costly than STB under current coverage, effectiveness, and average unit costs. On the other hand, the sensitivity analysis highlighted that the findings across all analyses were influenced by changes in effectiveness, unit costs, and coverage conditions. Scenarios with higher effectiveness and lower unit costs showed the most favorable CER and ICER values. This suggests that implementation efficiency and intervention quality are critical to achieving good economic value. When comparing the best and worst scenarios between STB and sealant, sealant showed a lower CER and provided more caries-free children, but at a higher cost than STB, representing a trade-off between additional effectiveness and additional cost. Sealant requires higher labor and material costs as a professionally applied resin-based procedure, while STB relies on teachers or parents supervising daily toothbrushing with fluoride toothpaste. Decision-makers should balance effectiveness and resources, using STB as a low-cost option and sealant as a more efficient or trade-off strategy for additional caries-free children when willing to pay.

This study has certain limitations. Not all caries-related factors, such as socioeconomic and behavioral variables, were incorporated into the model due to its complexity. The background characteristics and behaviors of the study population were assumed to reflect current conditions in Thailand (2). The model assumed that interventions reduce the rate of caries development by addressing risk factors and promoting oral health behaviors. The potential influence of factors such as parental guidance or individual behavior was considered. If these factors have a substantial impact on oral hygiene and dental care, the actual reduction in caries may be greater than estimated by the model. Conversely, if their impact is minimal, the estimated outcomes would remain largely unchanged. Thus, this structural uncertainty may not fully capture real-world variability. Given the data limitations, some parameter values were drawn from relevant literature, while most of the model’s values were derived from national and provincial databases in Thailand, and cost data were sourced from Thailand-based literature relevant to the model parameters. Effectiveness data were obtained through a systematic review and meta-analysis conducted by researchers (19, 32), and all data were validated by experts during GMB sessions. However, since parameters particularly rely on secondary data sources, parameter uncertainty may reflect potential variation in estimates of the model. Moreover, since the study focused on clinical outcomes, specifically the number of caries-free children, rather than quality-adjusted life years (QALYs), the findings may have limited comparability with economic evaluations in other healthcare areas that commonly use QALYs.

There are previous studies that applied SDM to evaluate the economic implications of oral health interventions. A study in the United States used SDM to evaluate the costs and impacts of multiple early childhood caries interventions (13). It showed that combined preventive strategies could substantially reduce costs and cavity prevalence over time. In Brazil, SDM has also been used to estimate the cost and workforce requirements for caries preventive programs and reported the usefulness of SDM in planning resources and costs (41). A study conducted in Thailand reported that SDM estimated costs of caries preventive programs and reported that the treatment costs due to caries were lower where preventive interventions were implemented (42). These studies are consistent with the present study, demonstrating that SDM can be used in economic implications and evaluating the oral health impacts of caries preventive interventions to support decision-making in oral health policy.

Conducting an SDM-based study aims to explore long-term projections, highlighting the importance of comparing different options, policies, or strategies. This study demonstrated that implementing caries preventive interventions, such as STB and sealant application, can reduce treatment costs for caries among primary school children over time. While sealant application incurs a higher average unit cost per child, making it more expensive than STB, STB remains a less costly intervention despite its comparatively lower effectiveness. From a policy perspective, it could help decision-makers in resource allocation and strategic planning for oral health initiatives. Since sealants offer greater clinical effectiveness, their high unit cost may limit nationwide implementation. It may inform decision-makers to prioritize high-risk populations or to plan a strategy with available resources to optimize cost-effectiveness and achieve broader oral health benefits for the population. Overall, SDM proves to be a valuable approach for gaining meaningful insights in this area.

In conclusion, both STB and sealant improved caries-free children compared to the base case, but sealant provided better value for money. Sealant had a lower CER than STB [505.3 baht (15.79 USD) and 537.2 baht (16.79 USD) per caries-free child] and a lower ICER [1,210.5 baht (37.83 USD) and 1,985.3 baht (62.04 USD) per additional caries-free child]. Sealant was dominant, with a negative ICER [−62.7 baht (−1.96 USD)], indicating it was more effective and less costly than STB under current conditions. In the best-and-best scenario of STB and sealant in sensitivity analysis, sealant was more effective but also more costly than STB, representing a trade-off option that provides a small additional cost per additional caries-free child compared to STB [ICER 121.54 baht (3.80 USD)]. From a policy perspective, these findings suggest that sealant is the preferred strategy or can be used as a trade-off option when decision-makers are willing to pay for additional caries-free children, while STB may serve as a lower-cost alternative in resource-constrained settings. Implementation should consider available resources, feasibility, and integration with existing oral health programs. Sealant should be prioritized by improving effectiveness while controlling costs to provide better economic value. Future strategies should focus on enhancing intervention efficiency and targeting high-risk populations to further improve cost-effectiveness.

## Data Availability

Publicly available datasets were analyzed in this study. This data can be found at: Data were obtained from the Health Data Center (HDC), Ministry of Public Health, Thailand.
