# Supplementary material for: Cost-effectiveness of 6-year school-based caries preventive interventions in Thailand: a system dynamics modeling study
Source: Front Public Health. 2026 May 14;14:1744986. doi: 10.3389/fpubh.2026.1744986 (PMC13215990; doi:10.3389/fpubh.2026.1744986)
Supplement: Supplementary file 1 [file Data_Sheet_1.pdf]

Supplementary Table S1 Parameters used in model

No.	Model parameter	Unit	Initial parameter value	Source	Assumption	Extracted data
1.	Population with caries-free	Person	436,817	Literature (SDM study) [19] and National statistical office (NSO) [25]	Used the 6-year-old population with caries-free from the previous related SDM study and the total population is from 2021 born (NSO source).	Caries-free = 436,817 (Literature)<br>Population = 678,243 (NSO)
2.	Population with untreated caries	Person	130,873	Literature (SDM study) [19] and National statistical office (NSO) [25]	Used the 6-year-old population with caries-free from the previous related SDM study and the total population is from 2021 born (NSO source).	Caries = 130,873 (NSO)<br>Population = 678,243 (NSO)
3.	Population with missing teeth	Person	44,086	8th National oral health survey (NOHS) [2] and National statistical office (NSO) [25]	Used the % of the closet 5-year-old population with missing teeth from the NOHS source and calculate the initial parameter value based on the total population of 2021 born (NSO source).	Missing teeth = 6.5% (8th NOHS)<br>Population = 678,243 (NSO)
4.	Population with filling	Person	59,685	8th National oral health survey (NOHS) [2] and National statistical office (NSO) [25]	Used the % of the closet 5-year-old population with filling from the NOHS source and calculate the initial parameter value based on the total population of 2021 born (NSO source).	Filling = 8.8% (8th NOHS)<br>Population = 678,243 (NSO)

5.	Population with endodontic treatment	Person	6782	Thailand literature (Pulpotomy 2563) [27] and National statistical office (NSO) [25]	Used the % of the >6-year-old population with endodontic from the literature and calculate the initial parameter value based on the total population of 2021 born (NSO source).	Endodontic treatment = 1% (Literature)<br>Population = 678,243 (NSO)
6.	Caries development fraction	Dmnl/Year (Dmnl=dimensionless)	0.14 (Base case)	Thailand literature (Oral health promotion foundation) [28]	Used the % of caries development from the control group after a 1-year follow-up from the literature and convert it to a fraction divided by 100.	Population with caries development = 14% (Literature)
			0.126 (Supervised Toothbrushing, STB for 6-12 years old)	Meta-analysis [32] and Thailand literature (Dental health promotion project 2548-2550) [29]	Meta-analysis is done to retrieve the effectiveness of STB for 6-12 years old. Caries development fraction was reduced by applying the effectiveness rate of STB after adjusted with coverage.	Coverage = 95% (Dental health promotion project 2548-2550)<br>Effectiveness of STB = 10% (Meta-analysis)<br>Thus, 0.1 * 95% = 9.5%<br><br>Caries fraction = Base case caries fraction × (1 – 9.5% /100)
			0.118 (Sealant 5-15 age intervals)	Meta-analysis [32] and Health Data Centre (HDC) [26]	Meta-analysis is done to retrieve the effectiveness of sealant for 6-12 years old. Caries development fraction was reduced by applying the effectiveness rate of sealant after adjusted with coverage.	Coverage = 27% (HDC)<br>Effectiveness of sealant = 58% (Meta-analysis)<br>Thus, 0.58 * 27% = 15.7%<br><br>Caries fraction = Base case caries fraction × (1 – 15.7% /100)

7.	Fraction of filling	Dmnl/Year	0.04	Health Data Centre (HDC) [26] and National statistical office (NSO) [25]	Used the number of populations with filling in the 2562 Buddhist year from the HDC source and calculate the fraction divided by the total number of populations in 2021 from NSO source.	Population with filling = 2,561,786 (HDC)<br>Total population (2021) = 66,171,439 (NSO)
8.	Fraction of recurrent caries from filling	Dmnl/Year	0.036	Literature (Secondary caries: prevalence, Ivana Nedeljkovic et al.,) [30]	Used the % of secondary caries from different types of restoration from the literature and convert it to a fraction divided by 100.	% of secondary caries = 3.6% (Literature)
9.	Fraction of endodontic treatment	Dmnl/Year	0.003	Health Data Centre (HDC) [26] and National statistical office (NSO) [25]	Used the number of populations with endodontic treatment in the 2562 Buddhist year from the HDC source and calculate the fraction divided by the total number of populations in 2021 from the NSO source.	Population with endodontic treatment = 163,419 (HDC)<br>Total population (2021) = 66,171,439 (NSO)
10.	Fraction of recurrent caries from endodontic	Dmnl/Year	0.006	Literature (Success or failure of endo Tx, Airton Oliveira Santos-Junior et al.,) [31]	Used the % of failure rate of RCT treatment and assumed that it is prone to recurrent caries from the literature and convert it to a fraction divided by 100.	% of failure rate = 0.6% (Literature)
11.	Fraction of missing teeth	Dmnl/Year	0.05	Health Data Centre (HDC) [26] and National statistical office (NSO) [25]	Used the number of populations with extraction in the 2562 Buddhist year from the HDC source and calculate the fraction divided by the total number of	Population with extraction = 3,525,836 (HDC)

					populations in 2021from the NSO source.	Total population (2021) = 66,171,439 (NSO)
12.	Unit cost of supervised toothbrushing	Baht/Person	57.6	Thailand literature [14,33-38] and Bureau of Trade and Economic Indices [39].	Cost estimates, study year, perspective, and cost components were extracted from each study and adjusted to 2024 price levels using the Thai Consumer Price Index (CPI; base year 2019 = 100) from the Bureau of Trade and Economic Indices.<br>Adjusted Cost 2024= Cost year × (CPI 2024) / (CPI year)	Average unit cost after adjusting = 57.6 in Thai baht
13.	Unit cost of sealant	Baht/Person	203.5	Thailand literature [14,33-38] and Bureau of Trade and Economic Indices [39].	Cost estimates, study year, perspective, and cost components were extracted from each study and adjusted to 2024 price levels using the Thai Consumer Price Index (CPI; base year 2019 = 100) from the Bureau of Trade and Economic Indices.<br>Adjusted Cost 2024= Cost year × (CPI 2024) / (CPI year)	Average unit cost after adjusting = 203.5 in Thai baht
14.	Unit cost of filling	Baht/Person	360.1	Thailand literature [14,33-38] and Bureau of Trade and Economic Indices [39].	Cost estimates, study year, perspective, and cost components were extracted from each study and adjusted to 2024 price levels using the Thai Consumer Price Index (CPI; base year 2019 = 100) from the Bureau of Trade and Economic Indices.<br>Adjusted Cost 2024= Cost year × (CPI 2024) / (CPI year)	Average unit cost after adjusting = 360.1 in Thai baht

15.	Unit cost of endodontic	Baht/Person	1839.4	Thailand literature [14,33-38] and Bureau of Trade and Economic Indices [39].	Cost estimates, study year, perspective, and cost components were extracted from each study and adjusted to 2024 price levels using the Thai Consumer Price Index (CPI; base year 2019 = 100) from the Bureau of Trade and Economic Indices.<br>Adjusted Cost 2024= Cost year × (CPI 2024) / (CPI year)	Average unit cost after adjusting = 1839.4 in Thai baht
16.	Unit cost of extraction	Baht/Person	133.6	Thailand literature [14,33-38] and Bureau of Trade and Economic Indices [39].	Cost estimates, study year, perspective, and cost components were extracted from each study and adjusted to 2024 price levels using the Thai Consumer Price Index (CPI; base year 2019 = 100) from the Bureau of Trade and Economic Indices.<br>Adjusted Cost 2024= Cost year × (CPI 2024) / (CPI year)	Average unit cost after adjusting = 133.6 in Thai baht
